# An assessment of influential factors developing the intention to use social media sites: A technology acceptance model based approach

**DOI:** 10.3389/fpsyg.2022.983930

**Published:** 2022-10-11

**Authors:** Tayyeba Bashir, Tan Zhongfu, Burhan Sadiq, Uzma Niaz, Faiza Anjum, Hassan Mahmood

**Affiliations:** ^1^School of Economics and Management, North China Electric Power University, Beijing, China; ^2^Department of Sociology, The Women University Multan, Multan, Pakistan; ^3^National Textile University, Faisalabad, Pakistan

**Keywords:** perceived usefulness, subjective norms, social media sites, efficiency, satisfaction, technology acceptance model

## Abstract

The aim of this study is to elaborate the different factors which attract users to use social media sites. These main factors are subjective norms, image, efficiency and satisfaction along with the mediating role of perceived usefulness. Survey method was used to collect data from B2B fashion brands in Pakistan. Convenient sampling technique was used to collect data from targeted respondents. Collected data was analyzed using Smart-PLS 3.3 version. Results express that subjective norms, image, efficiency and satisfaction have positive and significant impact on perceived usefulness and intention to use social media sites in B2B context within Pakistani fashion brands. Results further reveal that perceived usefulness partially mediates the relationship between subjective norms, image, efficiency, satisfaction and intention to use social media sites. This study will enhance the available literature in the area of technology acceptance model (TAM) and will provide useful insights to B2B managers to use efficiently use social media sites for the promotion of their brands. This study is limited to fashion brands, future researchers can expand this model to other area of business.

## Introduction

Over the course of the last 10 years, we have seen the development of social media into a multifunctional tool. The relevance of social media as a key marketing communication tool and as “a critical source of market knowledge” for B2B organizations has been recognized by business founders and developers, and firms are quickly implementing social media into their marketing strategy. Businesses need to adapt new technology if they want to be competitive in the future, which is expected to be dominated by digital media and online commercial transactions. As a result of the impact that information technology has had on the interactions that businesses have with their customers, managers have been looking for ways to gain a competitive advantage through digitalization (Kurdi et al., 2021).

According to a recent worldwide survey, business-to-business (B2B) marketers are hesitant to use social media platforms, despite the fact that B2B companies throughout the world are increasingly using social media. Research that was carried out in both the United States and Europe found that 55 percent of B2B organizations use social media sites to engage with colleagues both inside and outside the organization, whereas only 29 percent of B2B organizations incorporate social networks into their day-to-day operations (Choi and Chung, 2013). Additionally, the vast bulk of studies on the implementation and utilization of social media is focused on business-to-business companies from Western nations, which limits its application to economies that are still developing. According to Brennan and Croft (2012), the United States of America was the country that pioneered the use of social media in a business-to-business environment. It is difficult to ignore the growing reliance that business-to-business (B2B) companies have on social networking sites due to the prevalence of globalization and the widespread adoption of information and communication technology (Mohsin and Ivascu, 2022). This is especially true in today’s more global and dynamic business climate. As a result, businesses that cater to other businesses need to have a clear plan for how they will utilize social media and efficiently manage it. B2B companies that operate in developing nations are required to build effective social media strategies if they are to remain competitive in this era of increasing globalization and usage of social media (Aji et al., 2021).

There have only been a handful of studies done thus far on the usage of social media for business-to-business interactions in developing countries. For example, Pakistan has a 70 percent penetration rate for the internet and a 65 percent penetration rate for Facebook. Agag and El-Masry (2016) observed that the United States still trails behind in the integration of social media sites into their marketing strategy, which is contrary to what many B2B organizations have indicated. It is vital to map and understand the variables that inspire the utilization of social media platforms by B2B enterprises in developing nations. This is especially true in countries that are still developing economically.

It is not commonly recognized why businesses that cater to other businesses in developing nations use various kinds of social media. There has been a lot of study done on the usage of business-to-business social media in Western cultures, but there has not been as much research done on emerging markets and the characteristics that are more important there. Nielsen (1994b) asserts that efficiency is a crucial antecedent of technology adoption behavior, whereas Yeung and Law (2004) assert that usability is a combination of satisfaction and usability that leads to technology adoption. Both of these researchers believe that usability is the driving factor behind technology adoption. It was found that a person’s view of how simple it was to use, how advantageous it was, and how valuable it was to them came before their adoption of social media. The authors’ proposed paradigm places an emphasis on the factors that determine usability, which comes before the actual experience of usability by the user. Subjective standards, image, and the capacity to demonstrate results are major factors of perceived usefulness in the business-to-business (B2B) sector, where it is necessary to demonstrate accountability for outcomes and benefits.

According to a recent study, extending technology acceptance models and features of system acceptability models are utilized in order to provide an explanation for why B2B marketers in emerging nations use social networking sites.

This study will contribute in the existing body of literature in two ways which has been ignored in previous researchers. First, this study will bridge the gap in literature of technology acceptance model by adding the consumer behavior towards latest social media networking sites and this will enhance the readability of the new readers especially for the young readers. Second, this study will provide useful insights to the marketing professionals to attract more users towards their marketing pages on social networking sites and this will increase the flow of customers towards their pages. In previous researches most of the researchers only focused on technology acceptance model of consumer behavior separately, but in this research both the aspects have been combined and this combination will be useful for professionals as well as future researchers. Furthermore, this study follows others by articulating the elements that motivate B2B marketers in developing nations to use social media using the extended technology acceptance model (Venkatesh and Davis, 2000). On the basis of above discussion this study intends to answer the following questions:

RQ1: How does different TAM factors influence intention to use social networking sites?

RQ2: Do perceived usefulness mediates the relationship between different TAM factors and intention to use social networking sites?

This study is divided into three distinct parts. To get started, a literature review towards different factors that attract users to use social media sites Second, methodology is presented in terms of sampling, data collection and questionnaire development. After methodology, results section is presented. At the end the conclusion includes a discussion of the results, as well as its implications, limitations, and recommendations for further research are presented.

## Literature review and hypotheses development

### Technology acceptance model

Davis first introduced TAM in 1989, after having previously developed the “theory of reasoned action.” The Technology Acceptance Model (Davis, 1989) asserts that the mentality of customers toward technology is a significant component in determining whether or not technology is embraced and employed. People’s attitudes toward technology, their behavioral intentions to use technology, and, ultimately, their actual usage of technology are all impacted by how useful and easy people perceive it is to use.

When we talk about a system’s perceived ease of use, what we mean is “the degree to which a person believes that utilizing a certain system would be effortless,” as this is what we mean by “ease of use.” The perceived ease of use of a system is a measurement of how easy or difficult an individual perceives it to operate a system (Çelik, 2011). In terms of theory of action maturity, there are two primary reasons why people’s perceptions of how easy something is to use are so essential. To begin, an individual’s judgement of something’s usefulness has direct and indirect repercussions on their intents, and these repercussions are caused by the individual’s opinion of the something’s usability (Pratt et al., 2010). There is always going to be pushback from members of the public regarding any new technology since they have preconceived expectations regarding how easy it will be to implement and use (Verhoef and Bijmolt, 2019).

In contrast, the term “perceived usefulness” refers to the increases in productivity that can be achieved through the utilization of a particular piece of technology (such a computer). According to TAM, this is what happens when people evaluate different technologies: the more beneficial a technology is thought to be, the easier it is to use. This is one of the things that TAM believes happens when people evaluate different technologies (Weinberg, 2009).

Subjective norms are described by Ajzen (1991) as “a person’s judgement that the majority of his key individuals feel he should or should not conduct the behavior in issue.” Both the notion of reasoned action and the concept of planned conduct began with the contention that a person’s behavioral purpose may be directly influenced by the subjective standards that they hold (Ajzen, 1991). Venkatesh and Davis (2000) say that there is evidence to suggest that even if people do not want to do something, they will go along with it because it is required of them by external referents. This is the case even though there is evidence to suggest that people will do something even if they do not want to. An individual’s choice to behave in a particular manner may also be influenced by the individual’s social surroundings (Ajzen and Fishbein, 1975). In addition, research has demonstrated that an individual’s opinion of a technology’s utility has an indirect impact on whether or not they are willing to utilize that technology in situations when the usage of the technology is voluntary (Zhang and Li, 2019; Hussain et al., 2021). Subjective standards are also helpful in the early stages of social media adoption, when it is possible that users’ attitudes could be swayed by information. During this time, it is possible that users’ attitudes could be changed by information on the benefits of using social media. It was revealed that the benefit of sales acceleration that may gain from using online platforms was also found to have a major effect on subjective criteria (Buratti et al., 2018; Aji et al., 2021).

*H1*: Subjective Norms significantly affects perceived usefulness of social media sites

*H6*: Subjective Norms significantly influence intention to use social media site

Moore and Benbasat (1991) gave the following definition of social capital: “the extent to which the application of an idea is regarded to increase one’s status in one’s social system.” At this point in time, it was discovered that people’s judgments of the utility of new technology were greatly impacted by visuals (Karampela et al., 2020; Cartwright et al., 2021; Mohsin et al., 2021). It is essential to keep in mind that customers’ impressions of the prestige and status that come with utilizing a certain technology are directly influenced by the image associated with that technology. According to Bruhn et al. (2012) the image of a business has a significant influence on how customers see the usefulness of its use of social media. As a consequence, Jensen Schau and Gilly (2003)concluded in their study that B2B marketers regard social media sites as having a higher value when they have a good opinion of the medium’s utilization.

*H2*: Image of social media site significantly affects perceived usefulness

*H7*: Image of social media site significantly influence intention to use social media site

The capacity of a user to recall how to make use of social media is one of the factors that Nielsen (1994b) considers when evaluating the usability of a user interface. Lacka and Chong (2016) have shown that the ease with which social media networks may be remembered has a significant effect on the extent to which people value those platforms. In addition, the writers made the point that remembering social media use is rather simple. According to Nielsen (1994a), one of the most important aspects of usability is effectiveness. Nevertheless, Lacka and Chong (2016) discovered that the perceived utility of social media platforms in the context of business-to-business interactions is considerably and adversely influenced by the efficiency of those platforms.

According to Kumar et al. (2020), satisfaction may be satisfying the criteria and expectations of its users. According to Nielsen (1994a), the level of satisfaction experienced by end users is an essential component in determining the effectiveness of a technology. It is essential, as stated by Arpaci (2020), to differentiate between hedonistic and utilitarian values in order to have an understanding of the reasons why people make use of technology. In addition, Izuagbe et al. (2019) believe that pleasure-seeking reasons impact social media use and participation in the workplace. Mouakket (2015) claim that the level of enjoyment a user derives from a technology can be traced to both its interface and its content.

*H3*: Efficiency of social media site significantly affects perceived usefulness

*H4*: Satisfaction towards social media site significantly affects perceived usefulness

*H8*: Efficiency of social media site significantly influence intention to use social media site

*H9*: Satisfaction towards social media site significantly influence intention to use social media site

The degree to which a person feels that utilizing the system will boost his or her work performance, perceived simplicity of use, and the degree to which he or she believes that using the system will be easy all contribute to the system’s perceived utility (Amjad et al., 2018; Sullivan and Koh, 2019; Chatterjee and Kar, 2020). The perceived value of social media sites may be quantified, according to research conducted by B2B companies on industrial and IT organizations, by analyzing the amount of communication that is promoted and the number of appropriate audiences that are reached. According to Sobaih et al. (2022), there is a clear connection between the value that users consider social networking services to have and the extent to which such services are utilized by those users. B2B marketers are increasingly turning to social networking sites due to the perceived usability of these platforms (Dwivedi et al., 2015, 2021; Diba et al., 2019; Gul et al., 2021a). This perception is mostly impacted by the benefits of using these platforms, such as the capability to find new business prospects, boost sales, and enhance website traffic. Restrictions such as time and training in addition to a low perceived benefit, have a negative impact on the desire of B-to-B marketers to use social media. Lacka and Chong (2016) discovered that there is a substantial link between B-to-B marketers’ assessment of the utility of social media sites and their willingness to utilize those sites. This was one of the findings of their study. The key reason why B2B marketers utilize social media in the first place is to connect with new consumers and give current information (Li et al., 2021). According to Awan et al. (2021), B2B marketers regard social media sites to be beneficial since these sites allow them to interact with new clients (Veldeman et al., 2017; Masadeh et al., 2022). It has also been demonstrated that the propensity of B2B marketers to utilize social media is closely connected with their assessment of the utility of social media.

*H5*: Perceived usefulness of social media site positively influence intention to use social media site

Users analyze the performance of a technology to determine whether or not it meets their requirements. This is a perfect example of what we mean when we talk about “perceived usefulness.” Consumers evaluate the perceived utility of a technology in light of the application for which it was designed, as prior research has proven to be the driving factor behind the adoption of that technology (Gul et al., 2021b). As a consequence of this, the degree to which one believes a certain technology to be useful has a direct bearing on how much one wants to make use of that technology (Iankova et al., 2019; Chowdhury, 2022).

The user’s perception of how easily a product may be utilized is an essential component of the whole experience. There is a significant correlation between how people assess the practicability of a technological advancement and the likelihood that they will actually use it. According to Agnihotri (2020), it is unclear whether or not perceived usability and perceived usefulness are directly connected to one another. In a B2B context, Sharma (2021) found a correlation between the perceived usability of social networking platforms and the perceived utility of those platforms. According to Karampela et al. (2020), the perceived value of social networking sites is also based on how marketers see the simplicity of the sites. It has been discovered that the ease of use of social networking sites contributes to an increase in users’ trust, desire, and intention to utilize the services (Sullivan and Koh, 2019; Ding et al., 2022). To add insult to injury, if employees feel that social networking sites are more difficult to use than they actually are, they will evaluate the value of these platforms as being lower. The correlation between perceived usability and perceived usefulness is not as robust as the one that (Lacka and Chong, 2016; Diba et al., 2019; Mustafa et al., 2022). When it comes to promoting their goods and services to B2B customers, B2C businesses typically choose not to use social media since these sites are difficult to understand and use efficiently (Buratti et al., 2018; Zhang and Li, 2019; Jamil et al., 2022). This could be due to the fact that social media sites allow for two-way conversations between businesses and their customers. Therefore, following hypotheses are proposed:

*H10*: Perceived usefulness has mediating affect between subjective norms and intention to use social media site

*H11*: Perceived usefulness has mediating affect between image of social media site and intention to use social media site

*H12*: Perceived usefulness has mediating affect between efficiency of social media site and intention to use social media site

*H13*: Perceived usefulness has mediating affect between satisfaction towards social media site and intention to use social media site

Table 1 shows the brief summary of existing studies available on intention to use social media sites.

**Table 1:** A summary of existing social media studies.

**Studies**	**Cultural & Industrial Context**	**Independent Variables**	**Dependent Variable**	**Mediator**	**Moderator**
Arpaci (2020)	Turkish University Students	Social Interaction, Subjective Norms	Behavioral Intention	Attitude	N/A
Chowdhury (2022)	Bangladeshi Students	Attitude Subjective Norms Perceived Behavioral Control	Intention to use	N/A	N/A
Ding et al. (2022)	Chinese Consumers	Certainty Severity Celerity	Behavioural Intention	Subjective Norms	N/A
Irshad et al. (2020)	Pakistani Consumers	Consumer Motivations	Online Purchase Intention	Trust	N/A
Izuagbe et al. (2019)	Nigerian Consumers	Subjective Norms Voluntariness	Perceived Usefulness of Social Media	N/A	N/A
Lacka and Chong (2016)	UK Consumers	Learnability, Efficiency, Memorability, Errors, Satisfaction	Intention to Use	Perceived Usefulness	N/A
Yuan et al. (2021)	Malaysian Students	Social Media Literacy	User’s Attitude	Intention to use Social Media	N/A
Zhuang et al. (2021)	Chinese Consumer	Consumer Perception	Intention to Use	Attitude	Millennials vs. Non-Millennials
Agag and El-Masry (2016)	UK Consumer	Consumer Perception	Positive WOM	Intention to Participate	Religiosity
Aji et al. (2021)	Indonesian Consumers	Perceived Usefulness Ease of Use	Intention to use	E-Money	Knowledge of Riba

### Conceptual framework

**Figure 1:**
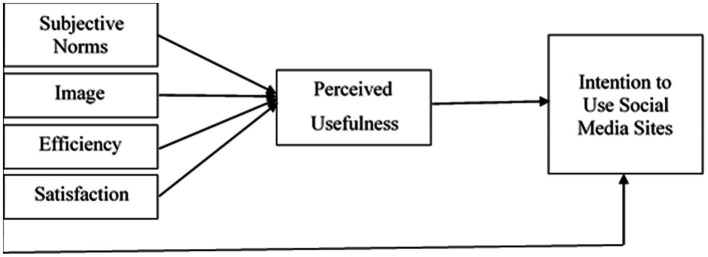
Conceptual framework.

Following Figure 1 indicates the conceptual framework and shows all relationships.

## Research methodology

### Research philosophy

Survey questionnaires were used for this study. When dealing with a sizable cross-section of a specific population, this strategy is efficient and practical. According to Rasool et al. (2020), researchers should design a survey before conducting any data collection. Similarly, a questionnaire was developed at the outset of this study to gather data from participants.

### Data collection process

Executives from fashion garment manufacturing organizations were approached to take part in the study. These executives include brand managers, product managers, vice presidents, and sales managers. Irshad et al. (2020) also conducted research on a local fashion market that was analogous to this one in the past. In addition to this, we obtained information from organizations of both a medium and a high level. According to the Small and Medium Enterprises Development Authority (SMEDA), a firm is considered to be medium if it has more than 100 workers, while it is considered to be big if it has more than 250 employees. That has more than one hundred people working on it. There is another school of thought that holds that a firm is deemed medium if it has more than one hundred employees. The respondents had an average of 12.6 years of expertise in managing brands, but only 8.5 years of experience in managing the brand they were currently working with. The sample size was taken into consideration, and the research team used a sample size formula that is both well regarded and widely used around the globe. The method focused on a limited population and was provided by Krejcie and Morgan (1970). In addition, in order to improve the extent to which the findings may be generalized, 450 questionnaires were sent out to respondents through internet poll between March 2022 and May 2022. A total of 430 responses were obtained, of which only 405 were considered valid and could be utilized; this yielded a response rate of 94.18%.

### Instrument development

To acquire a clear picture of the current relationship between the latent variables, researchers undertook an exhaustive literature review. The questionnaire consisted of 33 items and was created by integrating features from several studies. On a scale from 1 (strongly disagree) to 5 (strongly agree), respondents were asked to score each statement (strongly agree).

Perceived usefulness was measured with items adapted from the study of Lacka and Chong (2016) on a five-point Likert scale of one (strong disagreement with the item) to five (strong agreement with the item). The scale consisted of five items each.

Subjective norms were assessed with two items adapted from the study of Venkatesh and Davis (2000) on a five-point Likert scale of one (strong disagreement with the item) to five (strong agreement with the item). Image was consisted of three items adapted from Venkatesh and Davis (2000) efficiency consisted of two items and satisfaction was measured with three items both are adapted from Lacka and Chong (2016).

## Data analysis

### Model measurement

This study adopts a partial least squares modelling method instead of other covariance-based techniques like LISREL and AMOS. We chose PLS-SEM because it is best suited for exploratory and confirmatory research (Hair et al., 2016a,b). Structural equation modelling (SEM) comprises two techniques, namely covariance-based and partial least square SEM (Hair et al., 2016a,b). PLS is generally used to validate hypotheses, whereas SEM is more useful in hypothesis expansion. A PLS-SEM-based approach would be done in two parts, first weighing, and then measuring (Hair et al., 2014). PLS-SEM is excellent for a multiple-order, multi-variables model. To undertake tiny data analysis is equally beneficial in PLS-SEM. PLS-SEM provides it straightforward to compute all parameter computations (Awan et al., 2022; Hair et al., 2016a,b). The current study was done using Smart PLS 3.9.

**Figure 2:**
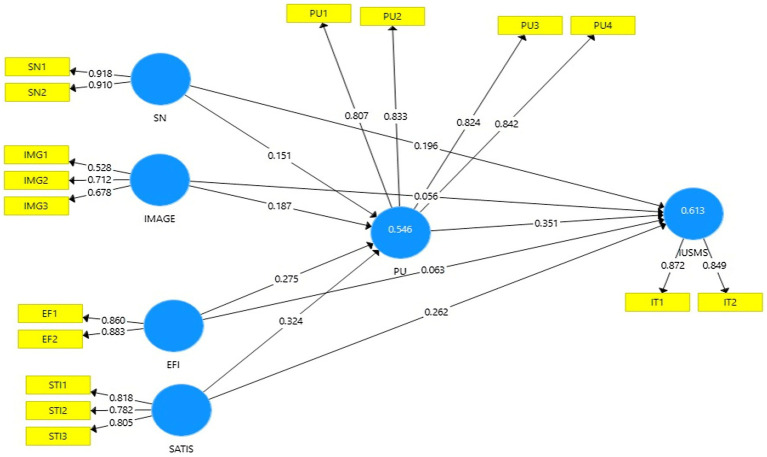
Measurement model.

**Figure 3:**
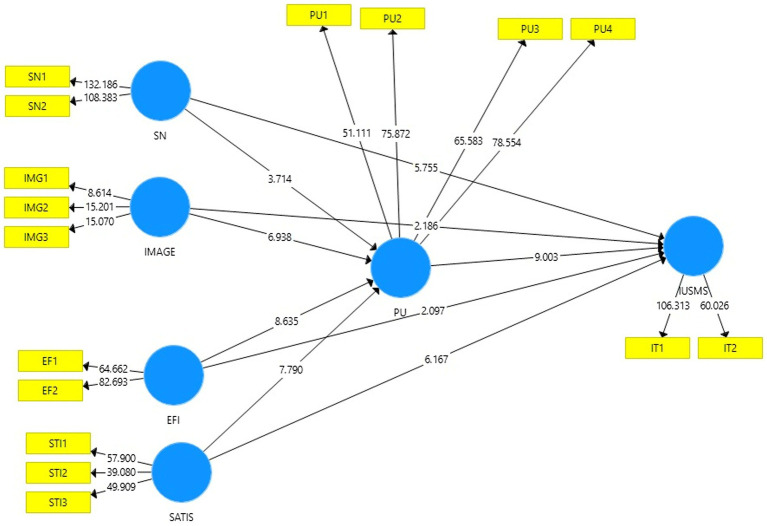
Bootstrapping.

Analysis of the model for measuring latent variables illustrates how dimensions of latent variables are respected in relation to their measurement qualities and perceived (observed) items. Assessment of the outer model (measurement) is done by looking at the internal consistency, item reliability, discriminant validity and convergent reliability of the measurement items (Hair Joe et al., 2016). There are total of 17 items in the model to help describe its six constructs. For all reflecting constructs, the PLS algorithm was used. The SMART PLS method was used to evaluate the convergent reliability and discriminant validity of the reflective scale. Latent variables (circles) and the items used to measure them are shown in the model in Figure 2, as shown below. According to Table 2, this research model includes According to Table 2, this research model includes Cronbach’s alpha is used to assess the research model’s dependability (Hair et al., 2016a,b). Table 2 shows that 11 items were deleted in order to increase the reliability of the items, and as can be seen, all items had strong loading and a Cronbach’s alpha more than 0.7 for all constructions. The composite reliability (CR) ranged from 0.767 to 0.910, above the allowed limit of 0.70 (Chin, 1998), indicating that all loadings utilized in this study had a sufficient indicator reliability (Chin, 1998; Baig et al., 2020). In the end, all items had loadings greater than or equal to the 0.6 criterion (see Figure 3).

**Table 2:** Inner model evaluation.

		**Item loading**	**Cronbach’s Alpha**	**rho_A**	**CR**	**AVE**
**Efficiency**	EF1	0.860	0.786	0.788	0.864	0.760
	EF2	0.883				
**Image**	IMG1	0.528	0.796	0.703	0.767	0.615
	IMG2	0.712				
	IMG3	0.678				
**Intention to use social media sites**	IT1	0.872	0.751	0.753	0.851	0.741
	IT2	0.849				
**Perceived usefulness**	PU1	0.807	0.845	0.846	0.896	0.683
	PU2	0.833				
	PU3	0.824				
	PU4	0.842				
**Subjective norm**	SN1	0.918	0.803	0.805	0.910	0.836
	SN2	0.910				
**Satisfaction**	STI1	0.818	0.722	0.724	0.843	0.642
	STI2	0.782				
	STI3	0.805				

In order to determine convergent validity, there are two methods: CR and AVE, and scale reliability for each item (Hair et al., 2016a,b). CR and AVE should be more than 0.7 and 0.5, respectively, according to a recent study. Coefficients of reliability and variance were used to evaluate scores’ converging validity. According to studies, as long as the composite reliability does not go below 0.70, it’s a good indicator of internal consistency (Hair et al., 2016a,b). Additionally, convergent validity is shown by average variance extracted scores higher than 0.50, which indicates that a specific construct with greater than 50% variability is explained by the necessary indicators (Chin, 1998).

Fornell–Larcker criterion is used to test discriminant validity (the ability to distinguish between two groups of people; Hair et al., 2016a,b). At least according to Fornell and Larcker (1981a), the square root of AVE, the model’s discriminant validity should be greater than the correlation with other variables, which is what these diagonal values show (Fornell and Larcker, 1981b; Hair et al., 2016a,b). It is shown in Table 3 which variable correlation with itself has the highest discriminant validity (Hair et al., 2016). A look at Table 3 confirms that the categorization used in this study showed discriminant validity are less than or equal to 0.90%.

**Table 3:** Discriminant validity.

	***EF***	***IMG***	***IU***	***PU***	***SAT***	***SN***
***EF***	0.872					
***IMG***	0.303	0.644				
***IU***	0.537	0.371	0.861			
***PU***	0.592	0.425	0.702	0.826		
***SAT***	0.560	0.339	0.698	0.658	0.802	
***SN***	0.522	0.295	0.657	0.599	0.770	0.914

Using R2 values for each predicted variable, we were able to determine the “explanatory power” of model. It demonstrates the extent to which independent variables depict dependent variables. R2 is between 0 and 1, with greater values indicating a better degree of prediction accuracy. R2 values range from 0.25 for “weak” to 0.50 for “moderate” to 0.75 for “substantial.” R2 > 0.5 indicates a suitable model prediction. Table 4 shows that all exogenous constructs have R Square values better than 0.613, which indicates a significant predictive accuracy for the model (Hair et al., 2016a,b).

**Table 4:** Predictive accuracy and relevance of the model.

Construct	R-Square (R^2^)	(Q^2^)
Intention to use	0.613	0.326
Perceived usefulness	0.546	0.449

Table 4 shows the proportion of variation that has been clarified for each variable. 61.3 percent of those surveyed said they had a desire to start their own business. A decent parsimonious model has R2 values less than 80 percent, but more than 50 percent, which is the case in the majority of cases. However, the results demonstrate the model’s robustness in a substantial way. Latent variable Q2 values indicate that the model is particularly predictive (Hair et al., 2014).

### Hypothesis testing

The findings show that Subjective Norms significantly affects perceived usefulness (*β* = 0.151, t-value = 3.714, *p* = 0.000). The findings show that Subjective Norms have significant impact on perceived usefulness (*β* = 0.187, t-value = 6.937, *p* = 0.000). the results illustrate that Satisfaction have significant impact on perceived usefulness (*β* = 0.324, t-value = 7.790, *p* = 0.000). the perceived usefulness has significant impact on intention to use social media site (*β* = 0.351, t-value = 9.003, *p* = 0.000).

The findings also show that Subjective Norms significantly influence intention to use social media site (*β* = 0.196, t-value = 5.755, *p* = 0.000). The Image of social media site significantly influence intention to use social media site (*β* = 0.056, t-value = 2.186, *p* = 0.000). Efficiency of social media site significantly influence intention to use social media site (*β* = 0.063, t-value = 2.097, *p* = 0.037). Satisfaction towards social media site significantly influence intention to use social media site (*β* = 0.262, t-value = 6.167, *p* = 0.000). The results of current study support the hypothesis H1, H2, H3, H4, H5 (see Table 5).

**Table 5:** Hypothesis testing.

		**Path Coefficient**	**Standard Deviation**	**T Statistics**	***P* Values**
H1	SN → PU	0.151	0.041	3.714	0.000
H2	IMAGE → PU	0.187	0.027	6.938	0.000
H3	EFI → PU	0.275	0.032	8.635	0.000
H4	SATIS → PU	0.324	0.042	7.790	0.000
H5	PU → IUSMS	0.351	0.039	9.003	0.000
H6	SN → IUSMS	0.196	0.034	5.755	0.000
H7	IMAGE → IUSMS	0.056	0.026	2.186	0.029
H8	EFI → IUSMS	0.063	0.030	2.097	0.037
H9	SATIS → IUSMS	0.262	0.043	6.167	0.000

### Mediation analysis

Perceived usefulness is the foundation upon which subjective norms, image satisfaction, and overall efficiency are built. Greater than 80 % of the variable indicates full mediation, greater than 20 % and greater than 80 % indicates partial mediation, and less than 20 % of the variable indicates there was no mediation. It has been shown that the sense of utility plays a mediating function in the connection that may be identified between subjective norms and the desire to utilize social media sites. There was an indirect impact (beta = 0.053, t-value = 3.473, value of *p* = 0.001) with VAF 68%, which indicates partial mediation. The findings indicate that perceived usefulness partially mediate the relationship between subjective norm and intention to use social media sites as the direct effect is significant (beta = 0.196, t-value = 5.755, value of *p* = 0.000) with VAF 57 percent, that indicates a partial balancing of powers VAF is a metric for quantifying the relative importance of direct vs. indirect effects, and it is expressed as a percentage. The findings suggest that perceived utility acts as a mediator between Image and social media usage inclination. There was an indirect impact (*β* = 0.066, t-value = 2.186, *p* = 0.000) with VAF 72 percent, which indicates partial mediation. The findings indicate that perceived usefulness partially mediate the relationship between subjective norm and intention to use social media sites as the direct effect is significant (*β* = 0.056, t-value = 2.186, *p* = 0.000) with VAF 78 percent, VAF is a measure of the indirect influence as a fraction of the total impact, implying that there has been some partial mediation (see Table 6). Both direct and indirect effects are minimized to some degree (Nitzl et al., 2016).

**Table 6:** Mediation analysis.

***Hypothesis***	***Direct effect***	***Indirect effect***	***Total Effect***	***VAF***	***Explanation***	***Result***
SN → PU → IUSMS	0.196 (5.755)	0.053 (3.473)	0.249 (6.230)	68%	Partial Mediation	H9 Supported
IMG → PU → IUSMS	0.056 (2.186)	0.066 (5.989)	0.122 (4.677)	57%	Partial mediation	H10 supported
EF → PU → IUSMS	0.063 (2.097)	0.097 (5.870)	0.159 (5.428)	72%	Partial mediation	H11 supported
SAT → PU → IUSMS	0.262 (6.167)	0.114 (5.506)	0.376 (8.235)	78%	Partial mediation	H12 supported

## Discussion of results

### Subjective norms and perceived usefulness

Researchers in Pakistan have made an important discovery on the relationship between favorable subjective standards and the perceived effectiveness of an application’s use of those standards. According to this research, the opinions held by B2B marketers on the significance, or lack thereof, of individuals using social networking sites influences their own usage of those sites, which is consistent with findings from past studies (Ajzen and Fishbein, 1975; Venkatesh and Davis, 2000). When it comes to this problem, B2B marketers in Pakistan and in other industrialized nations are in the same boat as one another.

### Image and perceived usefulness

The examination of the data showed that the design of social media websites has a significant influence on users’ perceptions of the usefulness of those sites. They believe that participating in activities on social networking websites will help them progress inside their company (Moore and Benbasat, 1991). In addition, the findings suggest that a greater awareness of the potential of social networking websites as instruments for effective marketing is connected with making an attempt to improve one’s image. Previous research conducted by Akbari et al. (2021) and Bruhn et al. (2012) found a significant correlation between the way something is shown and how it is judged to be useful. These findings suggest that businesses that promote to other businesses should incorporate social media sites into their marketing efforts. Marketers who make use of social media in the course of their work will view it as a means to advance the company’s objectives and will therefore be more likely to view it as a beneficial resource. This is because marketers who make use of social media in the course of their work view it as a means to advance the company’s objectives. In addition, the perceived worth of social networking sites is dependent on their image in developed nations, as the great majority of research that has been done in this area before has revealed (Bruhn et al., 2012).

### Efficiency and perceived usefulness

Moreover, B-to-B marketers have a substantial influence on how beneficial people thought social networking sites were viewed to be. As a direct consequence of this, the relationship in question was ignored. The research that Lacka and Chong (2016) conducted backs up this interpretation of their findings. If this is the case, then the efficiency of the tools that B-to-B marketers use in their personal life (for example, social networking sites) will be of greater importance to them than it is at B-to-B organizations, where efficiency is judged exclusively by personal usage (Nielsen, 1994b). On the other hand, the efficiency of social networking platforms does not appear to have an effect on the perceived utility of these platforms in the B-to-B market. The use of social networking sites is not anticipated to have any kind of positive effect on the performance or productivity of B-to-B marketers. Both of these studies are being conducted in developing nations at the moment. It has been shown that social networking websites in the Western world have a lower overall efficacy and are, as a result, less relevant for relationship-oriented usage than other business models. This was discovered through research conducted on B-to-B organizations (Gadhiya and Panchal, 2021). In addition, these results contradict the conclusions of a study that Nielsen (1994a) carried out in a developed nation and discovered that efficiency had an effect on usability. Nevertheless, additional social networking websites were also taken into consideration in the Nielsen analysis.

### Satisfaction and perceived usefulness

The data, which demonstrate a substantial association between a person’s perceived usefulness and their overall level of pleasure, lend credence to this viewpoint. These findings are not supported by the findings of Lacka and Chong (2016), who carried out research in China and discovered that there is no correlation between satisfaction and perceived usability. The attitude of the Pakistani culture, which is to take pleasure and success for granted, may be used to define the capacity to adapt to different social and professional settings (Yuan et al., 2021). However, satisfaction with the usage of social media sites does not alter Chinese B-to-B marketers’ perceived usability, and as a result, its function is not critical for the adoption of social media by Chinese enterprises.

### Perceived usefulness and intention to Use social media sites

There is evidence that the perspectives of business-to-business marketers on the usefulness of social media has an effect on their plans to use these platforms. As a consequence of this, marketers who consider social media to be a useful instrument are more likely to make use of it and acknowledge its value, and vice versa. This discovery is consistent with the findings of other investigations. For example, Lacka and Chong (2016) conducted research that provided empirical evidence that the propensity of Chinese B-to-B marketers to utilize social media sites is greatly influenced by their assessment of the utility of the sites. In a similar vein, Halim and Karami (2020) discovered that the perceived usability of social networking sites had a significant influence on the intents of South Korean B-to-B marketers to use them. Specifically, they found that the perceived usability of LinkedIn had a significant influence on the intents of South Korean B-to-B marketers.

### Theoretical implications

This conclusion has a wide range of implications for many theoretical frameworks. It makes a contribution to the research field of technology acceptance by combining two models: I the extended technology acceptance model and (ii) the model of the qualities of system acceptability. While Zhuang et al. (2021) suggested investigating characteristics that stimulate the use of social media platforms in a B-to-B environment, Lacka and Chong (2016) suggested assessing Nielsen (1994a) usability features in a new location. Both of these suggestions were made in response to a previous study that suggested investigating characteristics that stimulate the use of social media platforms in a B-to-C environment.

The capacity to show outcomes, image, and subjective criteria all have a large influence on perceived utility, which in turn has a substantial influence on the propensity of B-to-B marketers in developing nations to utilize social networking platforms. However, customer happiness and the number of mistakes made appear to have minimal impact on B2B marketers’ tendency to utilize social media. Effective usage of social media by B2B marketers tends to be comparable across nations. Additionally, the perceived utility of social media networks has an indirect impact on the desire of business-to-business marketers to use such networks. Customers will question if a piece of technology actually fulfils its intended function if it is not tied to the efficiency with which it does a task, as demonstrated by the example given above.

Insights into what motivates B2B marketers to use social media platforms are provided by this study’s novel, empirically proven methodology. This study shows that the level of social media site adoption and use for business-to-business marketing is influenced by marketers’ opinions of the sites’ usefulness, usability, and utility. In particular, our findings suggest that the adoption and utilization of social media sites for B2B marketing follows from marketers’ aspirations to do so. Our findings corroborate those of Nunan et al. (2018), who found that the likelihood of using social networking sites increases as one’s intention to do so grows stronger.

### Practical implications

Businesses that sell to other businesses should be more successful in their usage of social media in order to increase their marketing strategy and efficiency. An essential initial step is to have a deeper understanding of the reasons why business-to-business marketers opt to use social media platforms. As a consequence of these findings, it is feasible that replies to websites of this kind will have an impact on the patterns of adoption that they use. According to the statistics, B-to-B marketers are more inclined to utilize social media platforms if they regard them as beneficial. This is because social media platforms are becoming increasingly popular. On the other hand, the perceived usefulness and usability of social networking platforms are both determined by how easily the results can be demonstrated, the subjective standards they are held to, and their overall image. Moreover, the perceived usefulness of social networking platforms is influenced by how easily the results can be demonstrated. As a consequence of this, business-to-business companies might incentivize their staff to utilize social networking sites in their day-to-day operations by bestowing incentives on them for their participation in such activities. It is necessary to take into consideration the significance of a project’s potential to exhibit its outcomes in addition to the project’s image. Users of social media should be rewarded and appreciated, particularly by those working in the B-to-B business. In addition to this, it is important to recognize and acknowledge the efficacy of the usage of social media by B2B marketers. Invite people who are experts in social media to talk to the employees of businesses that sell to other businesses about the benefits of adopting social media into their day-to-day operations.

If B2B marketers want to start using social media for their campaigns, they must adjust their perceptions about the usability, usefulness, and utility of these platforms in the business world, according to the research shown above. Specifically, this study demonstrates that B2B marketers’ ambitions inspire the use of such sites for marketing purposes. Plans to utilize social media platforms for business-to-business marketing are closely correlated with their perceived usefulness, usability, and utility. Consequently, it is essential for marketers to have a more positive perception of social networking sites in order to promote their use and increase behavioral intentions. This can be conducted successfully after marketers have a more optimistic perspective of their own abilities to utilize social media sites, as well as evidence that these sites are, in fact, viable marketing channels *via* which B2B marketing objectives can be achieved. Therefore, we concur with Izuagbe et al. (2019), they recommended that “B2B firms should update their digital marketing use competencies.” To do this, it is essential to continually reinforce the utility of social media platforms in the business-to-business context. Instruction on the usage of social media platforms in business-to-business marketing is very beneficial for B2B marketers. As a result of social media marketing training, marketers’ perceptions of the value of social media platforms in business-to-business contexts will improve. This is due to the study’s conclusion that the learnability and memorability characteristics are responsible for the marketers’ perception of usability. Last but not least, it is important to note that marketers do not appear to have lofty objectives for business-to-business marketing *via* social media. Rather, our data indicates that marketers are receptive to embracing and utilizing social networking sites despite the potential inefficiency with which they may carry out particular marketing duties. Since marketing professionals do not place a high value on the effectiveness of social media marketing, B2B organizations may choose to reconsider their strategy.

## Conclusion

In this study, we build a testable framework by combining two existing models: Technology Acceptance Model (TAM) and Nielsen (1994b) Model of the Attributes of System Acceptance. This method is intended to assess the convenience with which marketing professionals utilize social media sites. This is accomplished by filling in the gaps in the existing literature regarding the factors that inspire individuals to join social media networks. In order to test the research framework and achieve the study’s objectives, we develop a survey questionnaire for marketers who utilize social media sites for promotional purposes. For instance, the evidence indicates that marketers are more likely to utilize social networking platforms for marketing objectives if they go out with that intention. Therefore, this result confirms Ajzen and Fishbein (1975) assertion that behavioral intentions are reliable predictors of technology uptake and use (e.g., social media sites). Moreover, our data indicate that the perceived value of social media sites by marketers has a major impact on their intended usage. This is consistent with the TAM-established notion. The likelihood that a marketer will use social media for promotional objectives is influenced by their familiarity and confidence with the platform, according to our research. However, the findings do not support the premise that marketing professionals’ perceptions of a technology’s usability have any effect on that technology’s effectiveness.

### Limitations and future research directions

There are three potential issues that might affect the reliability of these results. To begin, there is a very small number of people in the sample. It is possible that the data processing will be hampered by this sample size, which is equivalent to that of past studies. The data was given by Pakistan, and some of the conclusions reached were similar to those reached by other growing nations. Second, the most significant outcome of the research was that the efficiency of social networking sites did not have an impact on the perceived utility of those sites. Other findings ran counter to the conclusions drawn from the earlier research. When it comes to the usability of social media, it was discovered that B-to-B marketers are highly affected by inaccuracy and satisfaction, however B-to-C marketers are not impacted in any way by these factors. It would be beneficial to collect data from other emerging nations if one wanted to have a better understanding of the reasons why B-to-B marketers in emerging nations are more likely to utilize social media. Thirdly, prior to conducting the research, this study did not take into account perceived utility. In research on the elements that have an effect on social media use in both developed and developing countries, the factors that come before perceived usefulness can be investigated.

## Data availability statement

The raw data supporting the conclusions of this article will be made available by the authors, without undue reservation.

## Author contributions

All authors listed have made a substantial, direct, and intellectual contribution to the work and approved it for publication.

## Conflict of interest

The authors declare that the research was conducted without any commercial or financial relationships that could be construed as a potential conflict of interest.

## Publisher’s note

All claims expressed in this article are solely those of the authors and do not necessarily represent those of their affiliated organizations, or those of the publisher, the editors and the reviewers. Any product that may be evaluated in this article, or claim that may be made by its manufacturer, is not guaranteed or endorsed by the publisher.
